# Mn(II) Complex with Rutin—Spectral Characteristic, Quantum-Chemical Calculations, Antioxidant and α-Amylase Inhibitory Activity

**DOI:** 10.3390/ma19071466

**Published:** 2026-04-06

**Authors:** Maciej Kozłowski, Monika Kalinowska, Mariola Samsonowicz, Grzegorz Świderski, Beata Kalska-Szostko

**Affiliations:** 1 Department of Chemistry, Biology and Biotechnology, Institute of Civil Engineering and Energetics, Faculty of Civil Engineering and Environmental Science, Bialystok University of Technology, Wiejska 45E Street, 15-351 Bialystok, Poland; m.samsonowicz@pb.edu.pl (M.S.); g.swiderski@pb.edu.pl (G.Ś.); 2 Faculty of Chemistry, University of Białystok, Ciołkowskiego 1K 1, 15-245 Białystok, Poland; kalska@uwb.edu.pl

**Keywords:** phenolic compounds, rutin, antioxidant, metal complexes, antioxidant activity

## Abstract

Rutin is a naturally occurring flavonoid with well-documented antioxidant and pharmacological properties. In this study, a manganese(II) complex with rutin (Mn(II)-Rut) was synthesized in a solid state and characterized using FT-IR, Raman spectroscopy, thermogravimetric and elemental analysis, confirming its composition as C_27_H_27_O_16_Mn_2_·5H_2_O. The IR spectra indicated that rutin coordinates manganese ions through the carbonyl group at the C4 position and the hydroxyl group at the C5 atom, as well as the catecholic system. The antioxidant potential of both Mn(II)-Rut and rutin was evaluated using several spectrophotometric assays. The Mn(II)-Rut complex showed stronger activity in most spectrophotometric assays than rutin, i.e., in ABTS assay, 50.37 ± 2.64% vs. 41.49 ± 1.38%; in CUPRAC assay, 0.468 ± 0.006 mM Trolox vs. 0.379 ± 0.007 mM Trolox; and FRAP assay, 0.201 ± 0.002 µM vs. 0.189 ± 0.003 µM. However, the DPPH assay complex showed a diminished effect compared with ligand (IC_50_ 2.78 ± 0.13 µM vs. 0.98 ± 0.04 µM for rutin). Quantum-chemical calculations were also performed using the Gaussian09 program to determine the optimized geometric structures, electron charge distribution, and the energies of the HOMOs and LUMOs in the analyzed molecules in order to discuss the antioxidant mechanism of the molecules. Enzymatic assays demonstrated that the Mn(II) complex with rutin exhibited a stronger α-amylase inhibitory effect compared to free rutin, which showed the potential antidiabetic activity of the compound. The results suggest that the Mn(II) complex of rutin possesses better antioxidant and α-amylase inhibitory activity than the ligand alone.

## 1. Introduction

In today’s world, where the prevalence of civilization diseases such as cardiovascular diseases, cancer, diabetes, and neurodegenerative disorders is increasingly observed, compounds of plant origin are being studied more frequently. This trend stems from their wide range of biological activities, low toxicity, and antioxidant properties, which neutralize free radicals and inhibit oxidative stress—one of the key risk factors for many civilization diseases [1,2]. One such compound is rutin (quercetin-3-rhamnosyl glucoside), which belongs to the flavonoid group. Structurally, rutin is a flavonol quercetin linked to disaccharides: rhamnose and glucose [3]. Rutin was first discovered in buckwheat in the 19th century. The daily consumption of rutin usually varies between 1.5 and 70 mg/kg, depending on individual dietary habits [4]. Rutin occurs in a wide variety of plant-derived foods, although its concentration varies considerably depending on the species and plant part. Particularly high levels have been reported in buckwheat products, where rutin content ranges from 7.8 mg/100 g d.w. in noodles to 270 mg/100 g d.w. in leaf flour, while groats and dark flour contain approximately 23 and 21.8 mg/100 g d.w., respectively [5]. Capers are also considered a rich source of this flavonol, with rutin concentrations reported at 1.8 and 2.76 mg/100 g d.w. in buds and leaves, respectively, and up to 280 mg/100 g d.w. in fruits [6]. Moderate amounts have been detected in other plant foods such as amaranth (2.45 mg/100 g d.w.) [7] and radish (9.15 mg/100 g f.w.) [8]. Higher levels have also been reported in certain vegetables and herbs, including celery (417 mg/100 g f.w.) [9], cassava (622 mg/100 g f.w.) [10], lettuce (50 mg/100 g f.w.) [11], basil (15 mg/100 g f.w.), and coriander (115 mg/100 g d.w.) [12]. Exceptionally high rutin concentrations have been described in some fruits and herbs, such as apple (800 mg/100 g d.w.) [13] and tarragon (610 mg/100 g f.w.) [12].

Rutin demonstrates strong antioxidant activity, functioning as an effective free radical scavenger. This property is attributed to the hydroxyl groups attached to its aromatic rings. Additionally, rutin exhibits metal-chelating abilities, preventing metal ion-induced lipid peroxidation [14,15]. Its ability to scavenge free radicals has been proven through various assays, including hydroxyl, superoxide, DPPH, ABTS radical scavenging, and lipid peroxidation assays. Additionally, its metal-chelating activity has been confirmed in FRAP and CUPRAC assays [16]. Rutin has demonstrated powerful antioxidant capacity not only in vitro but also in vivo. In vivo studies revealed its significant antioxidant effects in diabetic rat liver, kidney, and brain after oral administration (100 mg/kg) for 45 days [17]. Researchers attribute the pro-health benefits of rutin to its potent antioxidant properties. These benefits stem from its diverse pharmacological activities, which include antiallergic, anti-inflammatory, vasoactive, anticancer, antibacterial, antiviral, and antiprotozoal effects. Furthermore, rutin demonstrates hypolipidemic and cytoprotective properties [15,16]. However, the health benefits of rutin can be influenced by its quantity and its bioavailability for absorption. The low water solubility of rutin (0.125 g/L) represents a limitation to its incorporation into functional foods and food supplements [18].

Rutin is a widely studied compound; however, its effectiveness and stability in the body can be enhanced by forming complexes with metal cations. Rutin has two potential chelation sites: 4-C=O/5-OH and the catecholic system. Samsonowicz et al. investigated the antioxidant potential of alkali metal salts of rutin. Their results indicate that the chelation of rutin by alkali metal cations influences its antioxidant properties. Among the tested salts, the rubidium salt exhibited the highest radical scavenging ability (DPPH assay) and FRAP, suggesting a potentially stronger protective effect against oxidative stress. In contrast, the sodium and lithium salts showed a reduction in their effectiveness in both assays [19]. Roy et al. synthesized vanadium complex of rutin and accessed its antioxidant properties using DPPH, FRAP and ABTS assays. The results they demonstrated showed that free rutin exhibited lower radical scavenging activity and ferric-reducing potential compared to the vanadium–rutin complex [20]. Panhwar et al. investigated the antioxidant capabilities of chrome (III) and tin (II) complexes of rutin using DPPH and FRAP assays. They found that complexes with chrome (III) exhibit higher antioxidant potency in both assays than rutin. However, the complex with tin (II) showed lower antioxidant capabilities than pure rutin [21,22].

In this paper, the manganese(II) complex with rutin was synthesized, and the structure of the compound was investigated using different analytical methods, infrared (FT-IR), thermogravimetric analysis for the solid samples, and mass spectrometry and electronic absorption spectroscopy (UV-Vis), for the solution. The antioxidant abilities of Mn(II)-Rut were assessed by spectrophotometric assay, FRAP (ferric-reducing antioxidant power), ABTS (2,2-azino-bis-3-ethylbenzothiazoline-6-sulphonic acid), CUPRAC (cupric reducing antioxidant power), DPPH (1,1-diphenyl-2-picrylhydrazil), and lipid peroxidation and compared with the rutin alone in order to establish the effect of metal ion coordination on the antioxidant properties of ligand. The molecular structure of rutin and Mn(II)-Rut was discussed on the basis of the theoretical calculations using Guassian 09 software. The calculated electronic parameters, including optimized geometric structures, electron charge distribution (via the NBO method [23]), and the energies of the HOMOs and LUMOs, were used to discuss the antioxidant activity of the studied compounds. In addition, enzymatic studies were conducted using the α-amylase inhibitory assay to evaluate their potential antidiabetic activity.

## 2. Materials and Methods

Rutin hydrate, potassium hydroxide, potassium bromide, trizma hydrochloride, trizma base, potassium persulfate, ABTS (2,2′-azino-bis(3-ethylbenzothiazoline-6-sulfonic acid) diammonium salt), DPPH (2,2-diphenyl-1-picrylhydrazyl), copper(II) chloride dihydrate, ammonium acetate, iron(III) chloride hexahydrate, trolox, sodium acetate, glacial acetic acid, TPTZ (2,4,6-tris(2-pyridyl)-s-triazine), ammonium thiocyanate, linoleic acid, Tween 60, iron(II) chloride tetrahydrate, α-amylase from Aspergillus oryzae, dimethyl sulfoxide, disodium hydrogen phosphate, sodium dihydrogen phosphate, and 3,5-dinitrosalicylic acid were purchased from Sigma-Aldrich (Saint Luis, MO, USA) and used without purification. Methanol, ethanol, and hydrochloric acid were purchased from Chempur (Karlsruhe, Germany).

### 2.1. Synthesis

**Figure 1:**
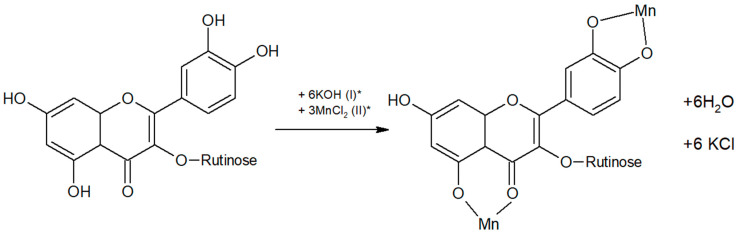
Proposed reaction scheme for the formation of the Mn(II)–rutin complex, where (I)* and (II)* refer to stages of reaction.

A measurement of 0.01 g of rutin was transferred into a test tube and dissolved in 4 mL of methanol. Subsequently, a stoichiometrically calculated amount of aqueous potassium solution (in a 1:1 molar ratio) was added to the prepared solution, followed by the addition of a manganese chloride solution in a 1:2 molar ratio. The test tube containing the mixture was then placed in a water bath at 80 °C and heated until a brown precipitate formed. The precipitate was then collected, filtered, and washed with methanol and distilled water. Reaction and proposed structure of complex is presented on Figure 1. The absence of free chloride ions was monitored during the washing process by testing the filtrate with AgNO_3_ solution. The washing procedure was continued until no formation of a white AgCl precipitate was observed, indicating complete removal of chloride ions. Then, it was placed in vacuum dryer for 12 h in temperature of 30 °C under pressure of 0.4 bar. Yield of reaction was 44%. The purity of the obtained Mn(II)–rutin complex was additionally verified by thin-layer chromatography (TLC). The analysis was performed on silica gel 60 plates coated with fluorescent indicator F254 (particle size 10–12 µm, layer thickness 200 µm; Supelco Bellefonte, PA, USA) in a glass chromatographic chamber using a solvent system composed of ethyl acetate, ethanol, and water in a volume ratio of 100:27:13. The chromatograms were developed for 30 min. The retention factor (Rf) value for rutin was 0.83 ± 0.029, whereas the Mn(II)–rutin complex exhibited a significantly lower Rf value of 0.30 ± 0.017. No spots corresponding to free rutin were observed in the chromatogram of the complex, indicating the absence of unreacted ligand and confirming the purity of the obtained compound.

### 2.2. FT-IR Spectra

The sample of the rutin and its manganese complex were placed in an agate mortar along with potassium bromide. The substances were mixed and ground together. The resulting powder was then placed in a hydraulic press to form a pellet. IR spectra were recorded over the range of 400–4000 cm^−1^ on Bruker ALPHA FTIR Spectrometer (Bruker, Billerica, MA, USA). The resolution was 4 cm^−1^.

### 2.3. Raman Spectra

To obtain Raman spectra of rutin and Mn(II)-Rut samples, a Renishaw InVia Raman spectroscope (Renishaw, Gloucestershire, UK) was used. The spectra were registered between 100 and 3200 cm^−1^. The investigation was performed using a semiconductor laser emitting 785 nm light. To record the spectra, a CCD detector with a resolution higher than 2 cm^−1^ was used. The laser beam was focused on the sample with ×20 objective.

### 2.4. NMR Spectra

NMR spectra were recorded on a Bruker Avance 400 spectrometer (Bruker, Billerica, MA, USA) operating at a basic frequency of 400 MHz for ^1^H spectra and 100 MHz for ^13^C spectra. Standard ^1^H, ^13^C, DEPT-90, and DEPT-135 spectra were acquired for samples dissolved in DMSO-d_6_ at room temperature (25 °C).

### 2.5. UV-Vis Spectra

Due to low solubility of the rutin in water and lack of solubility of its manganese complex in methanol, their solutions were prepared in water: methanol mixture (50:50, *v*:*v*) at 0.5 mM concentration. The solutions were then placed in quartz cuvettes, and their UV-Vis spectra were recorded over a wavelength range of 190–500 nm on Hach-Lange DR 5000 spectrophotometer (Hach-Lange, Loveland, CO, USA).

### 2.6. Mass Spectrometry

The sample was prepared by preparing two solutions of 10 mM concentration each, one of rutin and the other of manganese(II) chloride in methanol. The two solutions were then mixed in equal volume ratio and incubated for 30 min (after that time no precipitate occurred). After incubation, mass spectrometry coupled with a liquid chromatograph (Agilent Technologies 6420 Triple Quad LC/MS, Agilent Technologies, Santa Clara, CA, USA) was performed, but the column was removed. The following settings were applied: an *m*/*z* scan range of 50–2000. All analyzed samples were diluted in methanol, and the infusion rate was 5 µL/min. The spray voltage was set to 5.5 kV, and the capillary temperature was 180 °C. The fragmentor settings varied depending on the sample, and scans were performed in both positive and negative modes. The results were processed using Agilent MassHunter software B.07.00.

### 2.7. Thermal Analysis (TG-DCS)

Measurements were carried out on a TG 209 F1 Libra thermal analyzer (Mettler-Toledo, Columbus, OH, USA) under dynamic air flow at a rate of 20 mL/h. Samples weighing 10 mg were placed in alumina crucibles and subjected to heating from 25 °C to 930 °C at a constant rate of 10 °C/min. An elemental analysis to determine the carbon and hydrogen mass percentages was conducted using CHNS/O Elemental Analyzer (PerkinElmer, Waltham, MA, USA) in nitrogen atmosphere.

### 2.8. ABTS Assay

A rutin solution with a concentration of 0.01 M was prepared by dissolving an appropriately weighed amount of rutin in 5 mL of methanol in a 10 mL volumetric flask, followed by filling the flask to the mark with Tris-HCl (0.05 M, pH = 7.4). From this solution, two additional solutions with a concentration of 10^−5^ M were prepared using Tris-HCl for dilution. One solution contained rutin, while the other included a mix of rutin and manganese chloride in a 1:1 molar ratio. Solution of ABTS^•+^ was prepared by mixing in 1:1 ratio water solution of ABTS (C = 7 mM) and water solution of potassium persulfate (C = 2.45 mM); the mixture was left overnight in a dark place at room temperature. After this period, 1 mL of the working ABTS^•+^ solution was taken, and 60 mL of methanol was added to achieve a solution absorbance in the range of 0.7–1.0 at a wavelength of λ = 734 nm [24]. This absorbance was measured prior to performing the assay. The assay was conducted by adding 100 µL of the ABTS solution to a microplate, along with 50 µL of the test compound solution and 50 µL of Tris-HCl, ensuring a total mixture volume of 200 µL in each well. Measurements were taken over 15 min, during which the absorbance at λ = 734 nm was recorded. A mixture of 100 µL of ABTS solution and 100 µL of Tris-HCl was used as the control and Tris-HCl as blank. The antioxidant activity of the compounds (expressed as the percentage of ABTS^•+^ inhibition) was calculated using the following formula:

% inhibition = (A_control 734_ − A_test 734_)/A_control 734_

where A_control 734_ is the absorbance of the control sample without the addition of an antioxidant;

A_sample 734_ is the absorbance of the sample in the presence of an antioxidant.

All measurements were taken with six replicates, in three independent experiments on Infinite^®^ M Nano+ (Tecan, Männedorf, Switzerland).

### 2.9. DPPH Assay

Test solutions of rutin and its complex were prepared in the same way as in the ABTS assay. A methanolic solution of DPPH was prepared in a 25 mL flask by dissolving 0.0493 g of DPPH in methanol. From this solution, 3.36 mL was transferred to a 250 mL flask and diluted with methanol, resulting in a final concentration of 60 µM [25]. Assay was conducted by adding 200 µL of the DPPH solution to a microplate, followed by the test compound solution and Tris-HCl, ensuring a total well volume of 300 µL. Measurements were taken over the course of one hour, recording absorbance at a wavelength of λ = 516 nm. A mixture of 200 µL of the DPPH solution and 100 µL of Tris-HCl was used as the control and Tris-HCl as blank. The percentage of inhibition was calculated using the same formula as applied in the ABTS assay. The radical scavenging capacity was expressed as the EC_50_ value. All measurements were performed in triplicate for six replicates in independent experiments on Infinite^®^ M Nano+ (Tecan, Männedorf, Switzerland).

### 2.10. CUPRAC Assay

Copper(II) chloride was weighed and dissolved in distilled water to prepare a solution with a concentration of 10 mM. Ammonium acetate solution was prepared by dissolving it with water. Neocuproine was dissolved in ethanol to achieve a concentration of 0.075 M. The CUPRAC working solution was prepared by mixing the resulting solutions in a 1:1:1 volumetric ratio. A measurement of 3 mL of the CUPRAC working solution was transported into the test tube, followed by 0.5 mL of the test compound solution prepared as methanol/water solution (1:1) and 0.6 mL of distilled water [26]. Concentration of rutin and its complex in test tube was 10 µM. A blank sample was prepared by adding 0.5 mL of methanol/water solution instead of the test compound. The contents of the test tubes were mixed, sealed tightly, and incubated in the dark for 1 h. Subsequently, the absorbance was measured at a wavelength of 450 nm using a Nanocolor^®^ UV/VIS II spectrophotometer (Macherey-Nagel, Düren, Germany) against the blank sample. The experiment was conducted three times, with five replicates for each compound. The antioxidant activity was quantified as Trolox equivalents (mM Trolox; concentration range 0.05–0.5) using the calibration curve prepared for trolox (y = x × 1.8519 − 0.0707).

### 2.11. FRAP Assay

An acetate buffer with a pH of 3.6 was prepared by mixing sodium acetate with glacial acetic acid. A 20 mM solution of iron(III) chloride was then prepared by dissolving 0.5406 g in 100 mL of distilled water. Subsequently, a 10 mM TPTZ solution was prepared by 0.3155 g TPTZ, dissolving it in hydrochloric acid with a concentration of 0.04 M. Finally, a working solution was prepared by mixing the acetate buffer, TPTZ solution, and iron(III) chloride solution in a volumetric ratio of 10:1:1. Solutions of rutin and its metal complexes were prepared by weighing the samples and dissolving them in methanol/water solution (1:1) to achieve final concentrations of 50 µM and 10 µM respectively. The assay began by mixing 3 mL of the FRAP working solution with 0.4 mL of the methanolic solution of the test sample in a test tube [27]. The samples were then incubated at room temperature for 8 min. Subsequently, absorbance was measured at a wavelength of 595 nm using a Nanocolor^®^ UV/VIS II spectrophotometer (Macherey-Nagel). The experiment was conducted three times, with five replicates for each compound. The antioxidant activity was quantified in terms of Fe^2+^ equivalents (µM), based on a calibration curve constructed for FeSO_4_ in the range of concentration of 0.02–0.22 (y = 3.4382 × x + 0.0943).

### 2.12. Lipid Peroxidation Inhibition Capacity

To evaluate the inhibition of lipid peroxidation, several reagents were prepared. A 30% ammonium thiocyanate solution was obtained by dissolving 15 g of the compound in 50 mL of water. A 0.02 M iron(II) chloride solution in 3.5% hydrochloric acid was prepared by dissolving 0.1988 g of the hydrate in 50 mL of 3.5%. Additionally, an antioxidant solution with a concentration of 10^−3^ M was prepared in methanol/water solution (1:1). A linoleic acid emulsion was prepared in a 50 mL volumetric flask by combining 0.312 mL of linoleic acid and 0.256 mL of Tween 60, then filling the flask to the mark with 0.05 M phosphate buffer. For the assay, 1.5 mL of the linoleic acid emulsion was transferred into glass test tubes, followed by the addition of 1 mL of the methanolic solution of rutin and Mn(II)-Rut with concentration 0.5 mM [28]. Five replicates were prepared for each antioxidant in three independent experiments Control samples were prepared simultaneously, in which 1 mL of methanol was added instead of the antioxidant solution. The test tubes were sealed and incubated at 40 °C. Once the incubation temperature was reached, 0.1 mL of the mixture was taken from each tube and mixed with 4.7 mL of 75% methanol and 0.05 mL of the 30% ammonium thiocyanate solution. After waiting for 3 min, 0.05 mL of the 0.02 M FeCl_2_ solution in 3.5% HCl was added. The absorbance was measured at a wavelength of 500 nm using 75% methanol as a blank using a Nanocolor^®^ UV/VIS II spectrophotometer (Macherey-Nagel). The absorbance readings were taken every 24 h for five days. Each time, 0.1 mL of the same emulsion mixture was used for the measurement. The percentage of inhibition was calculated using the same formula as applied in the ABTS assay.

### 2.13. α-Amylase Inhibitory Assay

The evaluation of the inhibitory effect of rutin and Mn(II)-Rut complex was carried out using the method described by [24] with slight modifications. Rutin and its complex were dissolved in 70% DMSO to obtain solutions with appropriate concentrations in the range of 10–70 mg/mL. The highest concentration of the prepared solutions was 70 mg/mL, which was caused by the low solubility of the Mn(II)-Rut complex in DMSO. A measurement of 0.1 mL of rutin or complex solutions was mixed with 0.1 mL of 0.02 M phosphate buffer at pH 6.9 (with the addition of 0.006 M NaCl) containing enzyme (9 U/mL) and then incubated at 37 °C for 10 min. Then, 1 mL of 2% starch was added to each solution. After mixing, the reaction mixture was incubated for 10 min at 37 °C. The reaction was stopped by adding 1 mL of DNS, after which the mixture was incubated again in a boiling water bath for 10 min. After cooling, the reaction solutions were made up to 10 mL with distilled water. Absorbance was measured at a wavelength of 540 nm. The percentage of inhibition was calculated using the same formula as applied in the ABTS assay.

IC_50_ values were calculated from concentration–inhibition curves using nonlinear regression analysis of the dose–response relationship, based on at least seven concentration points within the tested range, and the results represent the mean values of three independent experiments.

### 2.14. Theoretical Calculations

The molecular structure of rutin and its complex with manganese(II) was investigated by quantum-chemical calculations using the Gaussian09 Revision D.01 software [25]. Visualization of the calculated parameters was performed using the molecular visualization program GaussView 6.0.9 [29]. The hybrid Density Functional Theory (DFT) method at the B3LYP/LanL2DZ level was used to determine the optimized geometric structures, electron charge distribution (NBO method [23]) in the analyzed molecules and energy of HOMOs and LUMOs. On the basis of the obtained HOMO and LUMO energy values, reactivity descriptors were calculated. Quantum-chemical calculations were performed in the gas phase to evaluate the intrinsic electronic structure of rutin and its Mn(II) complex, enabling direct comparison of key descriptors such as frontier molecular orbital energies and charge distribution without solvent-related perturbations. This approach was also justified by the use of multiple solvents in the experimental procedures, making a single implicit solvent model (e.g., PCM or CPCM) less representative of the system. Therefore, gas-phase calculations were considered a reasonable approximation for assessing fundamental electronic properties.

### 2.15. Statistical Analysis

All measurements were carried out in at least three independent experiments. The results are expressed as mean ± standard deviation (SD). The DPPH and ABTS assays were performed in six replicates in each of three independent experiments. The FRAP and CUPRAC assays were conducted in five replicates in each of three independent experiments. Lipid peroxidation assays were also performed in three independent experiments with five replicates per experiment. Statistical significance between rutin and Mn(II)-Rut in antioxidant assays was evaluated using Student’s t. Statistical significance has been defined at *p* < 0.05.

## 3. Results and Discussion

### 3.1. Studies for Solid Samples

#### 3.1.1. Elemental and Thermogravimetric Analysis

Based on the results of the elemental (Table 1) and thermal analysis (Table 2) the composition of the complex was determined as C_27_H_27_O_16_Mn_2_*5H_2_O. In the first stage of thermal decomposition of the complex, crystallization water is lost within the temperature range of 50–180 °C. The complex loses approximately 10.5% (11.1% theoretical loss) of its initial mass (five water molecules) at this stage. Coordinated water molecules bound directly to a metal center are typically released at considerably higher temperatures due to their stronger association with the metal ion.

A peak indicating an endothermic process is observed on the DTG curve, reaching a maximum at 79.8 °C. In subsequent stages of decomposition, rutin functional groups are lost and the complex structure destabilizes. Three peaks are observed on the DTG curve, with maxima occurring at temperatures of 239.2 °C, 412.8 °C, and 530.6 °C. The process was conducted in an inert gas environment (nitrogen), which resulted in incomplete combustion of the decomposition products. The process was terminated after reaching a temperature of 900 °C. The recorded mass loss was 59.2%. The residue from the process likely consists of manganese oxide and organic carbon residues.

#### 3.1.2. FT-IR and Raman Analysis

**Figure 2:**
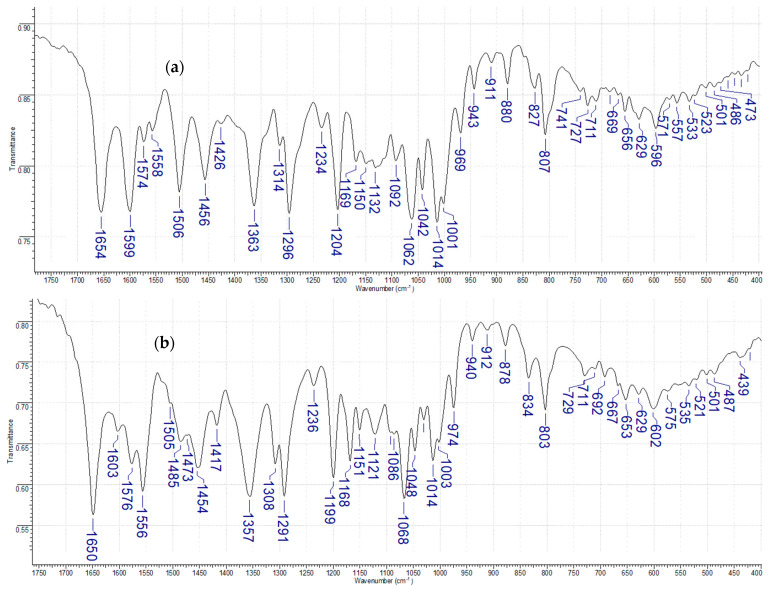
FT-IR spectra of rutin (**a**) and manganese(II) complex with rutin (**b**).

The chosen wavenumbers, intensities, and band assignments observed in the FT-IR (KBr and ATR solid) and FT-Raman spectra of rutin and its complex are summarized in Table 3. The assignment was based on the literature data [19,21,30,31]. In the KBr IR spectrum of rutin (Figure 2 and Figure S1), stretching vibrations of the hydroxyl group v(OH) originating from the aromatic ring were observed in the range of 3430 to 3073 cm^−1^. The bands at wavenumbers 2937 cm^−1^ v(CH), 2923 cm^−1^ v_as_(CH_2_), and 2923 cm^−1^ v_as_(CH_3_) are associated with the stretching vibrations of CH bonds in various hydrocarbon groups; corresponding vibrations are absent in Raman spectra. An intense band at 1654 cm^−1^ is attributed to the stretching vibrations of the carbonyl group v(C=O) with a Raman counterpart observed at 1678 cm^−1^. The bands at 1599, 1574, 1558, 1506, and 1456 cm^−1^ are assigned to the stretching vibrations of v(CC) in the aromatic ring for FT-IR spectra; for Raman spectra, 1637, 1576, 1550 1519 cm^−1^ bands were assigned. The asymmetric stretching vibrations v_as_(C–C–O) are assigned to the band at 1426 cm^−1^ and 1441 cm^−1^ for Raman. Bands corresponding to ν(C–O–C) and related ether vibrations are observed at 1363, 1296, 1204, 1062, and 1014 cm^−1^ in the IR spectrum, with Raman counterparts at 1383, 1318 and 1247 cm^−1^, respectively. The bands at 1169 and 1132 cm^−1^ are attributed to stretching and bending vibrations within the ketone moiety; Raman bands occur at 1194 and 1152 cm^−1^. Deformation vibrations γ(CH) and γ(C–OH) are assigned to bands at 807 and 628 cm^−1^ in IR and at 812 for Raman spectra.

In the spectra of rutin complexes with manganese(II), several changes were observed due to the coordination of the metal ion to the ligand structure. In the IR spectra of the complex, a shift in the ν(OH) stretching band toward higher wavenumbers (3431 cm^−1^) was observed.

The asymmetric stretching vibrations ν_as_(C–O–C) shift toward lower wavenumbers upon complexation. Bands observed at 1363 and 1296 cm^−1^ in free rutin shift to 1357 and 1291 cm^−1^, respectively, in the complex; corresponding Raman bands are detected at 1353 and 1225 cm^−1^. The band at 1132 cm^−1^, assigned to ketone stretching and bending vibrations, shifts to higher wavenumbers (1151 cm^−1^ IR; 1103 cm^−1^ Raman), indicating the involvement of the carbonyl group in coordination. In the spectrum of the manganese(II) complex, new bands at 497 cm^−1^ are recorded, originating from the bonding of the metal ion with the ligand. Based on this spectral evidence and the literature data [19,31,32,33], two binding sites are involved in metal coordination: (i) the carbonyl group at C4 and the hydroxyl group at C5 (4-C=O/5-OH chelation site, forming a six-membered chelate ring), evidenced by the shift in the ν(C=O) stretching band and the appearance of a new ν(Mn–O) band at 497 cm^−1^; and (ii) the catecholic system of ring B (3′,4′-diol), consistent with the observed shifts in the ν(C-O-C) asymmetric stretching and aromatic ring vibrations upon complexation. Since the spectroscopic data indicate coordination through both binding sites simultaneously, the total number of donor atoms from the rutin ligand alone precludes a simple geometric assignment without additional crystallographic evidence, which is not available for this compound.

### 3.2. NMR Spectroscopy

Attempts were made to record NMR spectra of the manganese(II) rutin complex. However, the obtained spectra were of low quality and not suitable for detailed structural interpretation (Figures S2 and S3). This is attributed to the paramagnetic properties of manganese ions. The presence of unpaired electrons in the manganese center induces strong paramagnetic relaxation effects, leading to significant signal broadening and reduced resolution. Consequently, reliable peak assignment and structural analysis based on NMR data were not feasible for this complex.

### 3.3. Studies of Methanolic and Aqueous Solutions

#### 3.3.1. UV-Vis Studies

**Figure 3:**
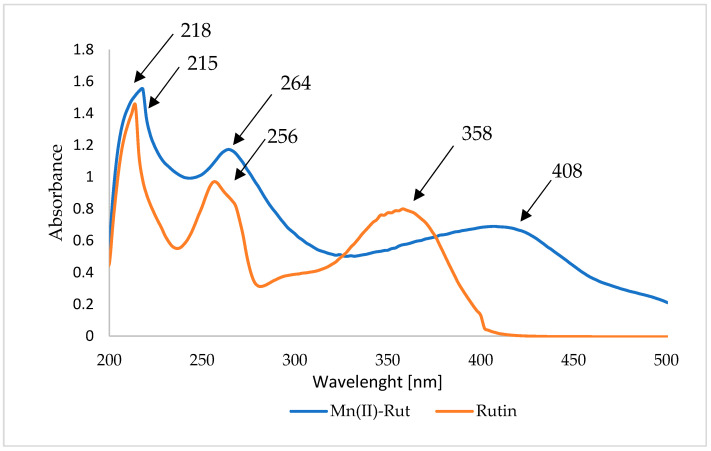
UV-Vis spectra of the methanolic water solution solutions of rutin and Mn(II)-Rut.

Rutin, like most flavonoids, exhibited two main absorption bands in the UV-Vis range, 200–400 nm [20]. The UV-VIS spectra of methanolic solutions of studied compounds were shown in Figure 3. Band I at 358 nm indicates the presence of a cinnamoyl moiety and phenolic -OH groups (associated with a conjugated system between the B ring and the carbonyl group of the C ring); band II at 256 nm is attributed to a benzyl moiety (associated with a conjugated system between the A ring and the carbonyl group of the C ring) [20,22]. In the spectrum of the manganese salt of rutin, we observe bathochromic shifts in bands II and I, confirming the formation of a complex. The absorption bands present in the UV-Vis spectra of rutin and its salts result from electronic transitions: n→π* (electron transfer from a non-bonding molecular orbital n to an anti-bonding molecular orbital π*, occurring in the C=O group with a lone electron pair) and π→π* (electron transfer within the aromatic ring) [32].

#### 3.3.2. Mass Spectrometry

**Figure 4:**
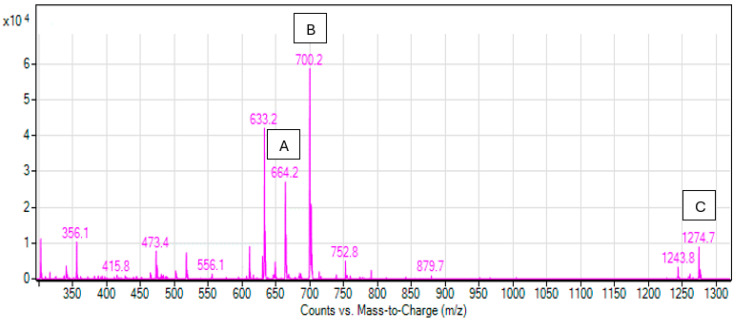
Mass spectra of Mn(II)-Rut complex, Peaks A, B, and C correspond to different forms of the complex: A = [(R–H)Mn]^+^, B = [(R–H)Mn + 2H_2_O]^+^, and C = [(2R–H)Mn]^+^.

The mass spectra of the manganese(II) complex (Figure 4) indicate the formation of complexes. For Peak A (*m*/*z* 664), B (*m*/*z* 700), and C (*m*/*z* 1274), based on the molecular masses, A corresponds to [(R–H)Mn]^+^, while complex C is [(2R–H)Mn]^+^ (molar ratio R/Mn = 2:1). Additionally, peak B at *m*/*z* 700 can be observed, which may potentially belong to the [(R–H)Mn]^+^ complex with two water molecules attached. Peaks at *m*/*z* 611, 633, and 1243, corresponding to rutin ([R + H]^+^), rutin with a sodium ion ([R + Na]^+^ and [2R + Na]^+^), were identified in the positive mass spectrum, while other peaks might correspond to various glucose fragments [20].

#### 3.3.3. Antioxidant Activity

The antioxidant potential of rutin and its manganese(II) complex was evaluated using several complementary assays: DPPH, ABTS, FRAP, and CUPRAC. Each of these assays provides insight into a different mechanism of antioxidant action, including free radical scavenging and metal ion reduction. Results are presented in Table 4. It should be noted that while the solid-state structure may involve different stoichiometries, mass spectrometry analysis indicates that the 1:1 Mn(II)–rutin complex is the predominant species in solution and is therefore considered the most relevant form for antioxidant activity.

In the DPPH assay, rutin exhibited stronger radical scavenging activity than the Mn(II)-Rut complex, with IC_50_ values of 0.98 ± 0.04 µM and 2.78 ± 0.13 µM. This assay is based on the reduction in the stable DPPH• radical in methanolic solution, resulting in a decrease in absorbance at 517 nm. The DPPH method primarily reflects a hydrogen atom transfer (HAT) mechanism, in which the availability and reactivity of phenolic -OH groups play a key role [34,35]. Coordination of Mn(II) may partially affect these groups, which can contribute to the lower activity of the complex compared to free rutin. Additionally, solvent effects may influence the observed activity. Rutin is more soluble in methanol, which is the primary solvent in the DPPH assay, whereas the Mn(II)-Rut complex shows higher affinity for aqueous media. However, due to the lack of quantitative solubility data, this factor should be considered as a contributing element rather than the sole explanation.

In contrast, the ABTS assay revealed slightly higher antioxidant activity for the Mn(II)-Rut (50.37 ± 2.64%) compared to rutin (41.49 ± 1.38%). This method involves the reduction in the ABTS^•+^ radical cation, which absorbs at 734 nm, and is predominantly governed by electron transfer (SET) mechanisms [36].

The FRAP assay, which evaluates the ferric-reducing antioxidant power, indicated marginally higher antioxidant activity for the Mn(II)–rutin complex compared to rutin at both tested concentrations. Specifically, the FRAP values for the Mn(II)–rutin complex were 0.201 ± 0.002 µM at 10 µM and 0.096 ± 0.004 µM at 5 µM, whereas for rutin they were 0.189 ± 0.003 µM and 0.079 ± 0.003 µM, respectively. This assay is based on the reduction in Fe^3+^ to Fe^2+^, forming an intensely blue Fe^2+^-TPTZ complex, and reflects electron transfer processes [37]. Similarly, in the CUPRAC assay, which monitors the reduction in the Cu(II)-neocuproine complex at 450 nm under near-physiological pH, the Mn(II)-Rut (0.468 ± 0.006 mM Trolox) exhibited higher reducing capacity than rutin (0.379 ± 0.007 mM Trolox). It should be noted that different solvent systems were used in the applied assays (TRIS HCl with methanol for DPPH and ABTS, methanol:water solution for FRAP and CUPRAC), which may influence solubility, reaction kinetics, and the relative contribution of different antioxidant mechanisms. In each assay, both compounds were tested under identical conditions, allowing direct comparison within a given method. However, differences observed between assays may partly arise from solvent effects combined with mechanistic differences between HAT- and SET-dominated pathways Coordination of Mn(II) with rutin may influence these mechanisms in several ways. Metal coordination can modify the electron density distribution within the flavonoid structure and affect the redox properties of the molecule. In particular, interaction with Mn(II) may facilitate electron transfer processes and stabilize radical intermediates through metal–ligand interactions [34,35].

**Figure 5:**
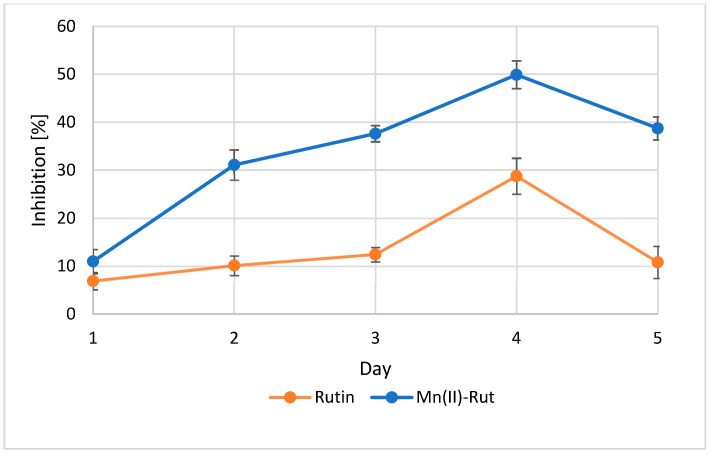
Lipid peroxidation inhibition assay of rutin and Mn(II)-Rut measured for five days. Values are expressed as mean ± SD. Statistical significance was determined using Student’s *t*-test (*p* < 0.05).

Lipid peroxidation is a chain reaction process involving the oxidative degradation of polyunsaturated fatty acids. The ability of compounds to inhibit this process is an important indicator of their antioxidant potential, particularly in biological systems where lipid oxidation contributes to oxidative stress (Figure 5). Both rutin and the Mn(II)-Rut complex reduced the rate of lipid oxidation throughout the incubation period. The complex demonstrated stronger inhibition, with a maximum inhibition rate of 49.9% on the fourth day, compared to 28.7% for free rutin under the same conditions. However, we can observe a decrease in the inhibition for both compounds on the fifth day.

Qadeer K. Panhwar and Shahabuddin Memon evaluated the antioxidant activity of rutin and its Cr(III)complex using DPPH, FRAP, and total antioxidant capacity assays. In their study, rutin exhibited high DPPH radical scavenging potential due to the catechol moiety on its B-ring, which stabilizes radicals via intramolecular hydrogen bonding. Authors suggested that the Cr(III) complex showed higher DPPH activity than free rutin because chromium coordination disrupted the intramolecular hydrogen bond, making the hydrogen at the 4-position more available for abstraction [22]. FRAP and total antioxidant capacity assays indicated that the Cr(III) complex with rutin slightly enhanced the electron-donating ability and overall redox potential compared to free rutin [22]. Ikeda et al. reported that the Zn(II) complex with rutin exhibits enhanced antioxidant activity relative to free rutin in both DPPH and superoxide radical (NBT) assays. The Zn(II) complex inhibited 80% of DPPH radicals at 30.4 μM, compared to 45% for rutin at the same concentration. Superoxide scavenging activity measured for the compounds at the concentration of 100 μM was also 25% higher for the complex than for free rutin [33]. Panhwar and Memon also studied the Sn(II) complex with rutin, finding a substantial decrease in the antioxidant activity of the complex relative to free rutin. DPPH inhibition dropped from 68% (rutin) to 26% (Sn(II) complex), and FRAP measurements were reduced by approximately half. This decrease was attributed to structural and electronic alterations induced by tin coordination, which hinder hydrogen abstraction and electron transfer [21]. Samsonowicz et al. also reported that complexation of rutin with different alkali metals alters its antioxidant activity. Specifically, complexes with potassium, rubidium, and cesium exhibited increased antioxidant activity compared to rutin, whereas lithium and sodium complexes showed lower activity in the DPPH assay. However, complexes with potassium and cesium exhibited different activity changes depending on the assay, increasing in DPPH and lowering in FRAP assays [19].

#### 3.3.4. Enzymatic Studies α-Amylase Inhibitory Assay

**Figure 6:**
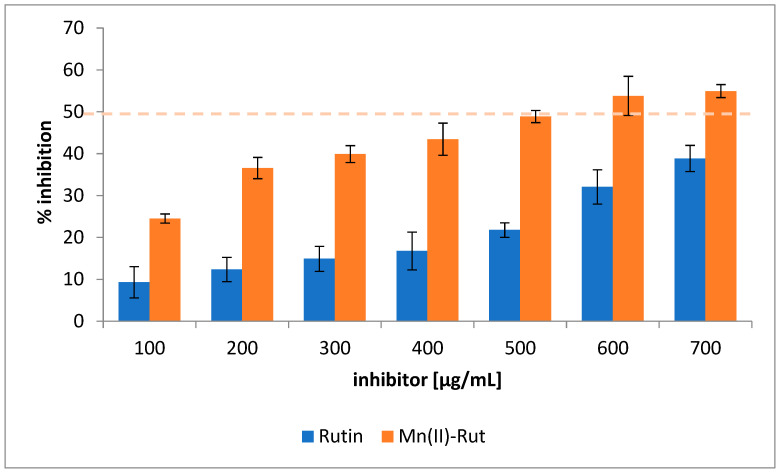
Inhibitory activity of rutin and Mn(II)-Rut. The results are expressed as mean ± standard error.

As shown in Figure 6, the rutin and Mn(II)-Rut complex exhibited a dose-dependent inhibition of α-amylase activity. The results obtained showed that manganese(II) complex has a higher α-amylase inhibitory activity than rutin under the tested conditions. The calculated IC_50_ values of rutin and its complex with Mn(II) were 0.771 and 0.542 mg/mL, respectively. The maximum inhibition rate of α-amylase reached 49.95% in the case of rutin and 54.95% in the case of the Mn(II)-Rut complex. The inhibitory potency of α-amylase inhibitors is strongly dependent on the experimental conditions, such as enzyme source and activity, substrate type and concentration, incubation time, temperature, pH, and solvent composition. These parameters can significantly influence enzyme kinetics and the interaction between inhibitors and the enzyme active site [38].

Enhancement in bioactivity through metal complexation is consistent with previous findings involving other flavonoid–metal systems. For instance, Dong et al. reported superior hypoglycemic, antioxidant, and antimicrobial effects of a luteolin manganese(II) complex compared to free luteolin, attributing the improvement to metal-induced stabilization of the flavonoid and enhanced interaction with biological targets [39].

The observed IC_50_ values differ from some previously published results for rutin. For example, Dubey et al. reported an IC_50_ value of approximately 250 µg/mL for rutin [40], while Oboh et al. found a lower IC_50_ of 0.043 µM, indicating variability in potency depending on assay conditions such as enzyme origin, reaction medium, and substrate type [41]. Aleixandre et al. demonstrated that the inhibitory potency of phenolic acids was significantly reduced in the presence of starch due to sequestration of the active compounds. Although this phenomenon was not evaluated in the present study, it suggests that real-food matrices could affect the bio efficacy of rutin and its complex. Notably, phenolics bearing more hydroxyl groups such as gallic and chlorogenic acid retained activity despite starch interference, underscoring the importance of hydroxylation patterns in enzyme inhibition [42,43]. The lower IC_50_ observed for the rutin Mn(II) complex provides compelling evidence that metal coordination may enhance the inhibitory activity of the flavonoid. Several mechanistic explanations can account for this effect. First, rutin’s polyphenolic structure, which includes multiple hydroxyl groups and sugar moieties, is known to facilitate hydrogen bonding and π–π stacking interactions with key residues in the α-amylase active site [43]. Upon complexation with manganese(II), the flavonoid’s geometry and electronic distribution are altered, potentially increasing its affinity for the enzyme through stronger electrostatic interactions or a better fit into the catalytic groove [39,44]. Furthermore, metal chelation may increase the compound’s resistance to metabolic degradation enhancing, which may contribute to improved biological activity [45].

### 3.4. Theoretical Calculations for Molecules in a Gas Phase

#### 3.4.1. Theoretical Structure

**Figure 7:**
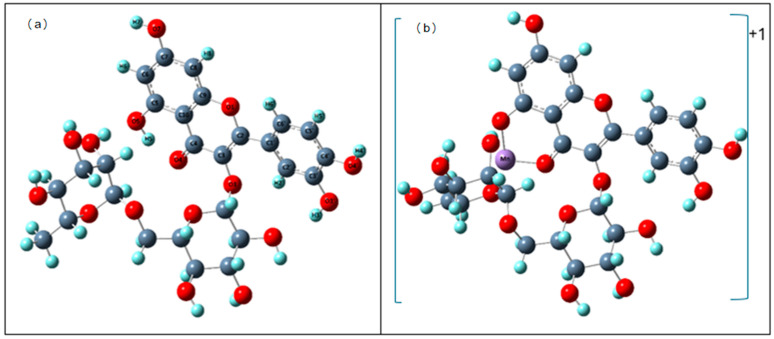
Optimized structures of rutin (**a**) (with atom numbering) and rutin–manganese cation (+1) complex (**b**).

The optimized structures at B3LYP/LANL2DZ of rutin and its Mn(II) complex in the gas phase are shown in Figure 7. The initial structure for calculations was the rutin structure presented by Samsonowicz et al. [19]. The primary goal of the quantum-chemical calculations was not to reproduce the solid-state structure of the complex, but to determine electronic parameters, including optimized geometry, NBO charge distribution, and HOMO/LUMO energies that are directly related to the chemical reactivity of the studied compounds, in particular their antioxidant properties. For this purpose, the 1:1 species is the most relevant model, as it represents the dominant form identified in solution by mass spectrometry (peaks at *m*/*z* 664 and 700, corresponding to [(Rut–H)Mn]^+^ and [(Rut–H)Mn(H_2_O)_2_]^+^, respectively) and is also the stoichiometry used in the preparation of solutions for all antioxidant assays. The gas-phase 1:1 computational model therefore provides a chemically meaningful basis for the discussion of reactivity which are the principal outcomes of the theoretical section (Figure 7). However, it should be noted that in biological systems these compounds operate in a solvated environment, and solvent effects may influence their stability and reactivity.

The main geometric data (bond lengths and angle sizes) of rutin and its complex with manganese are summarized in Table S1 (in the Supplementary Materials). The data indicate that the formation of the complex with manganese influenced the changes in bond lengths and angle sizes between atoms in the ligand molecule. Compared to the free rutin molecule, the most noticeable changes were observed in rings A and C (bond length changes exceeding 0.01 Å), whereas complexation did not affect the bond lengths in ring B. The C4–C10, C6–C7 and C5–O5 bond lengths decreased, while the C10–C9, C10–C5, C4–O4, and C5–C6 bond lengths increased. A similar trend was observed when analyzing the angle sizes. The largest modifications were observed in rings A and C for the following angles: C3–C4–O4, C4–C10–C9 and O5–C5–C6 (decrease by 4.85o, 2.60o, 2.02o, respectively) and C3–C4–C10, O4–C4–C10, C4–C10–C5, C10–C5–O5 (increase by 2.37o, 2.48o, 3.70o, 3.39o, respectively).

The NBO electron charge distribution was calculated for rutin and rutin–manganese cation (+1) complex molecules in the gas phase. The calculations are summarized in Table S2, whereas Table 5 shows the electronic charges on atoms that changed by more than 0.1e after the formation of the complex (atoms labeling is according to Figure 7). The formation of the complex resulted in an increase/decrease in electron density (by 0.01–0.02e) mainly around the atoms in the A and C rings adjacent to the site of attachment of the Mn(II) ion. The largest decrease in electron density compared to the free ligand was observed around the atoms C3, C10 and O4, while the largest increase was around the following atoms: C2, C4 (by 0.083), C6, C7, C8, O1, O5, O7 and H6.

#### 3.4.2. Molecular Electrostatic Potential

**Figure 8:**
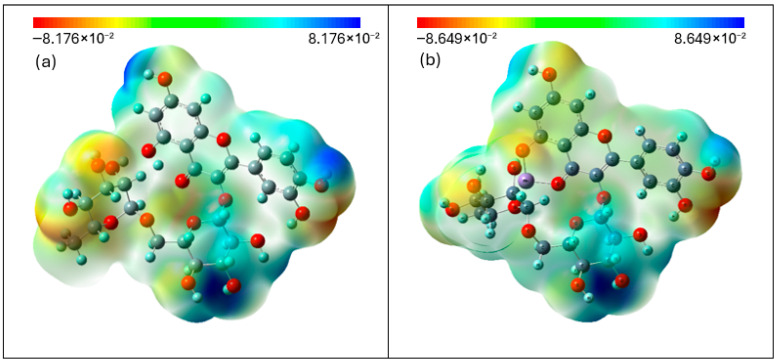
Generated molecular electrostatic potential (MEP) maps of rutin (**a**) and rutin–manganese complex (**b**). The negative (red, orange, and yellow) regions are related to electrophilic reactivity, whereas positive (green and blue) regions accompany nucleophilic reactivity.

Molecular electrostatic potential (MEP) is widely used to determine the most likely regions in a molecule susceptible to nucleophilic and electrophilic attacks [38]. The red or orange region indicates electron-rich sites (with the highest negative charge), which are associated with electrophilic reactivity, while the blue or green region indicates electron-deficient sites, corresponding to regions associated with electrophilic reactivity (Figure 8). The regions associated with electrophilic reactivity are associated with the carbonyl group (C=O) in ring C, the carbonyl group (C=O) within the C ring, the oxygen atom of the hydroxyl group at the C5 position of the C ring, and the oxygen atom of the hydroxyl group attached to the C3′ carbon in the B ring (Figure 7). Additionally, electrophilic character is observed around the oxygen atoms of hydroxyl groups present in the glucose moiety of the disaccharide linked to the aglycone. In turn, nucleophilic regions are predominantly found in the vicinity of hydrogen atoms from hydroxyl groups bonded to the C4′ (ring B) and C7 (ring A) carbon atoms, as well as near the carbon atom within the rhamnose unit of the disaccharide.

The formation of the Mn(II)-Rut did not result in significant changes in the spatial distribution of regions with negative and positive molecular electrostatic potential compared to rutin alone. Only changes in the maximum and minimum values of this potential were observed.

#### 3.4.3. HOMO–LUMO Analysis

**Figure 9:**
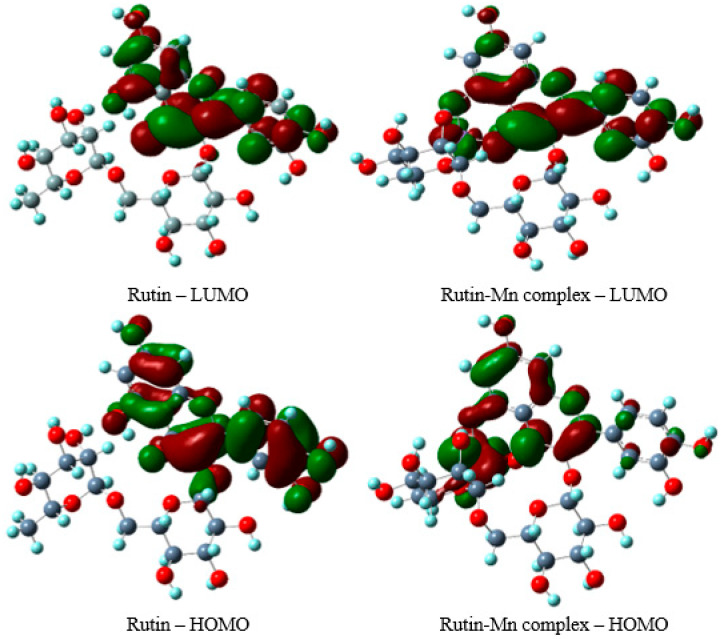
The shape and energy of the HOMO and LUMO molecular orbitals in rutin and its complex with manganese.

The energies of the Highest Occupied Molecular Orbital (HOMO) and the Lowest Unoccupied Molecular Orbital (LUMO) are critical parameters in quantum-chemical computations and serve as valuable indicators for the qualitative assessment of molecules’ reactivity and biological activity [46]. The HOMO energy reflects the electron-donating capability of a molecule, whereas the LUMO energy corresponds to its electron-accepting potential. In the context of antioxidant activity, HOMO energy is particularly significant, as it is directly associated with electron transfer mechanisms (Figure 9). Molecules exhibiting lower HOMO energies demonstrate reduced propensity to donate electrons during interactions with free radicals. Conversely, the higher the HOMO energy is, the greater the ability to carry out nucleophilic attacks is, facilitating more effective electron donation in redox processes [47]. The HOMOs and LUMOs of rutin and the complex are presented in Figure 8. In the rutin molecule, the HOMO is mainly located on ring B, while the LUMO is located on ring C. In the complex molecule, however, the HOMO is primarily found on manganese and rings A and C, whereas the LUMO is located on ring C. The computational results indicate that the HOMO energy of the Mn(II) cationic complex (+1) is lower (−4.0169 eV) than that of rutin (−6.2690 eV), suggesting that rutin exhibits a stronger electron-donating ability compared to the complex.

The HOMO–LUMO energy gap of compounds helps to predict their antioxidant potential and stability. A lower gap value corresponds to increased chemical reactivity, characterizing the molecule as “soft”. The slightly higher HOMO–LUMO gap observed in the Mn(II)-Rut complex suggests its higher stability (Table 6), whereas the lower gap in free ligand implies greater chemical reactivity compared to its complex. Based on the energies of the HOMOs and LUMOs, parameters used for evaluating the electronic properties of the analyzed molecules, such as ionization potential (I), electron affinity (A), electronegativity (χ), electronic chemical potential (µ), chemical hardness (η) and softness (S) and electrophilicity index (ω), were calculated and are summarized in Table 6. These parameters are closely related to the antioxidant strength of the molecules [48].

It was noted that the ionization potential value for the complex is lower than that of rutin, which may indicate an increase in the electron-donating properties of the complex in redox reactions compared to the ligand itself. Electron affinity measures the energy change when an electron is added to the molecule. The electron affinity of rutin (6.269 eV) was found to be higher than that of its manganese complex (4.017 eV), indicating a greater intrinsic tendency of the free ligand to accept electrons. Electronegativity (χ), which reflects the ability of an atom or molecule to attract electrons, was also higher for rutin (4.455 eV) compared to the Mn(II)-Rut complex (2.969 eV). This suggests a reduced electron-attracting capability upon complex formation. Chemical hardness (η) and chemical softness (S) are descriptors that provide insight into molecular stability and reactivity. The Mn(II)-Rut complex exhibited lower hardness (1.048 eV) and higher softness (0.477 eV) relative to the free ligand, implying enhanced chemical reactivity and a greater propensity to participate in chemical interactions. Finally, the electrophilicity index (ω), which characterizes a molecule’s tendency to accept electrons, was lower for the Mn(II)-Rut complex than for rutin. This observation indicates a diminished electrophilic character of the complex in comparison to the free ligand.

Solvation generally stabilizes polar and charged species and may influence electronic descriptors such as HOMO–LUMO energy gaps, charge distribution, and metal–ligand interactions [49]. In flavonoids, these effects are particularly relevant for antioxidant mechanisms involving electron transfer and proton dissociation, such as SET-PT and SPLET pathways [50]. Coordination of Mn(II) modifies the electron density distribution within rutin, affecting frontier molecular orbital energies and charge delocalization. The HOMO energy reflects the electron-donating ability, which is a key parameter in antioxidant activity, while charge redistribution (e.g., NBO analysis) provides insight into metal–ligand interactions and reactive sites. Similar relationships between electronic structure and biological activity have been widely reported for phenolic compounds [35,51].

## 4. Conclusions

Comprehensive spectroscopic, thermal, and elemental analyses confirm the successful synthesis of the manganese(II) complex with rutin in the solid state and provide insight into the structural modifications resulting from metal coordination. The complex was characterized as C_27_H_27_O_16_Mn_2_·5H_2_O, with the coordination of manganese ions occurring through the carbonyl group at the C4 position, the hydroxyl group at C5, and the catecholic system of the rutin molecule. Mass spectrometric analysis identified molecular ion peaks corresponding to 1:1 ligand:metal species as the dominant form in the solution. Theoretical DFT calculations supported the experimental results for rutin in Mn(II)-Rut with a metal:ligand ratio of 1:1. The calculated ionization potential value was lower for the complex compared to rutin, which may indicate an increase in the electron-donating properties of the complex in redox reactions compared to the ligand. The experimental data showed that the complex exhibits increased reducing power in CUPRAC and marginally in FRAP assays, improved ABTS radical scavenging compared to rutin, that indicates the possibility of its use as a natural-origin substance with antioxidant properties. Moreover, Mn(II)-Rut possessed greater α-amylase inhibition compared to free rutin. Our findings support the hypothesis that metal–flavonoid complexes, such as the rutin–manganese(II) complex, can act as more potent enzyme inhibitors than their parent ligands [39]. These results not only align with previous research but also reinforce the potential application of metal–flavonoid complexes in the dietary management of type 2 diabetes. It is well established that in vitro biological effects of flavonoid–metal complexes often correlate with their in vivo activity trends. Therefore, the observed results may suggest potential physiological relevance; however, further investigations, particularly in vivo studies, are required to confirm their translational significance and applicability.

## Figures and Tables

**Table 1:** Elemental composition of manganese complex with rutin.

Complex Formula	Exp. C Content [%]	Theor. C Content [%]	Exp. H Content [%]	Theor. H Content [%]
C_27_H_27_O_16_Mn_2_·5H_2_O	40.13 ± 0.23	40.12	4.82 ± 0.026	4.58

**Table 2:** Thermoanalytical results (TG, DTG) for manganese complex of rutin.

Complex Formula	Stage	TG T_range_/°C	DTG T_max_/°C	Mass Loss/%		Residue
				Calc	Found	
C_27_H_27_O_16_Mn_2_·5H_2_O	I dehydratation	50–180	79.8	11.10%	10.50%	C_27_H_28_O_16_Mn_2_
	II decomposition	200–900	239, 412.8, 530.6	-	59.2%	MnO, C_org_

**Table 3:** The wavenumbers [cm^−1^], intensity (Int.) and assignments of bands occurring in the experimental FT-IR and Raman spectra of rutin and Mn(II)-Rut.

Rutin				Mn(II)-Rut				Assignment
IR_KBR_	Raman			IR_KBR_		Raman		
cm^−1^	Int.	cm^−1^	Int.	cm^−1^	Int.	cm^−1^	Int.	
3425	vs			3431	vs			v(OH)_ar_ [21]
2938	m			-				v(CH) [19]
2923	m			2941	m			v_as_(CH_2_), v_as_(CH_3_)
1654	m	1678	s	1650	s	-		v(C=O) [21]
1599	m	1637	s	1602	m	1553	vs	v(CC) [30]
1574	m	1576		1576	m	1522	vs	
1558	m	1550		1556	s			
1506	m	1519		1485	m			
1456	m							
1426	w	1441	s	1417	m	1424	vs	v_as_(C–C-O) [30]
1363	m	1383	s	1357	s	1353	vs	v(C–O-C) [19]
1314	m			1308	m			v(C–OH) [19]
1296	m	1318	s	1291	s	1225	s	v(C–O–C) [29]
1234	w	1247	m	1236	m			v_as_(O-C-C) [30]
1204	m			1999	s			v(C-O-C) [31]
1169	m	1194	m	1168	m	1147	s	(C–CO–C) stretch and bending in ketone moiety [19]
1132	m	1152		1151	m	1103	m	
1092	m	1114	m	1086	m			v(C–OH) [19]
1062	m			1068	s			v(C–O-C) [31]
1014	m			1014	m			v(C–O–C) [31]
943	m			940	w			γ(CC)
807	m	812	m	803	m	812	m	γ(CH) [30]
628	w			629	m			γC–(OH) [30]
-				497	w			v(Me–O) [21]

s—strong; m—medium; w—weak; vs—very strong. The symbol “v” denotes stretching vibrations. “γ” denotes in-plane bending modes. “γ” designates out-of-plane bending modes. “as” means asymmetric; “ar’’ stands for aromatic.

**Table 4:** Comparison of antioxidant activity of rutin and its manganese complex. Values are expressed as mean ± SD. Statistical significance was determined using Student’s *t*-test (*p* < 0.05).

Assay [Units]	Concentration of Tested Compound	Rutin	Mn(II)-Rut	*p*
DPPH [IC_50_]/[µM]	-	0.98 ± 0.04	2.73 ± 0.11	<0.001
ABTS [%]	5 [µM]	41.49 ± 1.38	50.37 ± 2.64	<0.05
CUPRAC [mM Trolox]	10 [µM]	0.379 ± 0.004	0.468 ± 0.005	<0.001
FRAP [µM Fe^2+^]	10 [µM]	0.189 ± 0.001	0.201 ± 0.002	<0.01
	5 [µM]	0.079 ± 0.003	0.096 ± 0.003	<0.01

**Table 5:** Optimized structures of rutin (a) and rutin–manganese cation (+1) complex (b).

Atoms ^1^	Charge [e]	
	Rutin	Mn(II)-Rut
Ring B		
C4′	0.327	0.314
Ring C		
C2	0.375	0.345
C3	0.185	0.195
C4	0.470	0.387
C10	−0.245	−0.232
O1	−0.520	−0.537
O4	−0.644	−0.635
Ring A		
C6	−0.341	−0.354
C7	0.390	0.374
C8	−0.309	−0.325
O5	−0.760	−0.772
O7	−0.714	−0.725
H5/Mn	0.528	0.642
H6	0.240	0.228

^1^ The numbering atoms in the rutin molecule is shown in Figure 7.

**Table 6:** Chemical reactivity parameters of rutin and Mn(II)-Rut complex in vacuum solution obtained at the B3LYP/LANL2DZ level of theory in gas phase.

Parameter	Rutin	Mn(II)-Rut
Chemical reactivity parameters		
Energy (Hartree )	−2250.18	−2353.45
Dipole moment (Debye)	4.50	5.51
E_HOMO_ (eV)	−6.269	−4.017
E_LUMO_ (eV)	−2.642	−1.921
ΔE_(HOMO—LUMO)_ (eV)	3.627	3.910
Ionization potential, I = −E_HOMO_ (eV)	6.269	4.017
Electron affinity, A = −A = −E_LUMO_ (eV)	2.642	1.921
Electronegativity, $\chi =\frac{I+A}{2}$ (eV)	4.455	2.969
Electronic chemical potential, $\mu =-\frac{I+A}{2}$ (eV)	−4.455	−2.969
Chemical hardness, $\eta =\frac{I-A}{2}$ (eV)	1.814	1.048
Chemical softness, S $=\frac{1}{2\eta }$ (eV)	0.276	0.477
Electrophilicity index, $\omega =\frac{\mu^{2}}{2\eta }$(eV)	5.472	4.205

## Data Availability

The original contributions presented in this study are included in the article/Supplementary Material. Further inquiries can be directed to the corresponding authors.

## Supplementary Materials

The following supporting information can be downloaded at https://www.mdpi.com/article/10.3390/ma19071466/s1. Figure S1. Raman spectra of rutin and Mn(II)-Rut; Figure S2. 1H-NMR spectra of Mn(II)-Rut; Figure S3. 13C-NMR spectra of Mn(II)-Rut; Table S1: Selected bond lengths (Å) and angles (o) between bonds in rutin and rutin–manganese complex molecules; Table S2: NBO atomic charges1 on atoms in molecules of rutin and its manganese complex.
