# Supplementary material for: Mn(II) Complex with Rutin—Spectral Characteristic, Quantum-Chemical Calculations, Antioxidant and α-Amylase Inhibitory Activity
Source: Materials (Basel). 2026 Apr 6;19(7):1466. doi: 10.3390/ma19071466 (PMC13074668; doi:10.3390/ma19071466)
Supplement: Supplementary file 1 [file materials-19-01466-s001.zip › materials-4081886-supplementary.pdf]

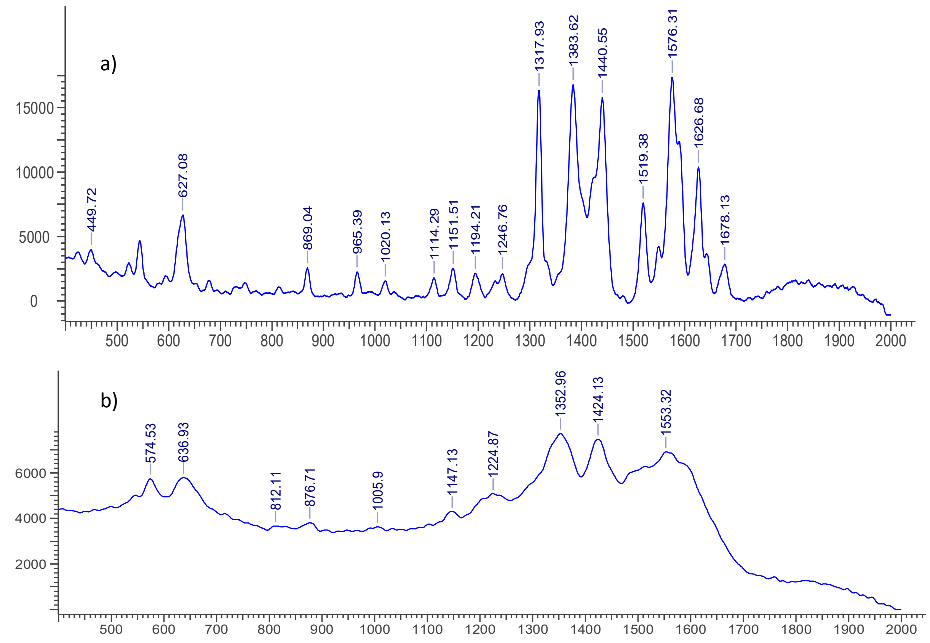

Figure S1. Raman spectra of rutin (a) and Mn(II)-Rut (b).

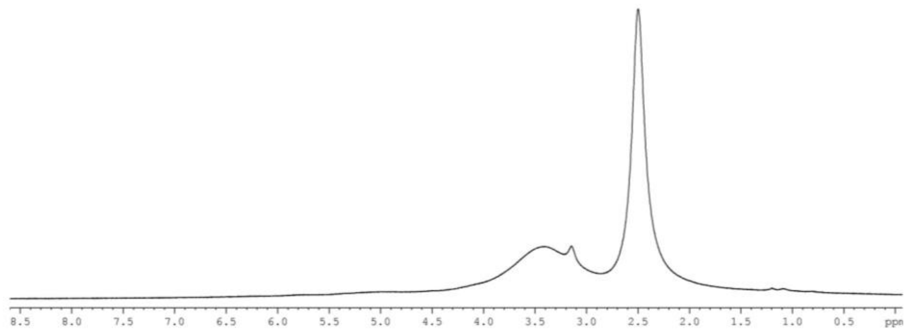

Figure S2. ^{1}H-NMR spectra of Mn(II)-Rut.

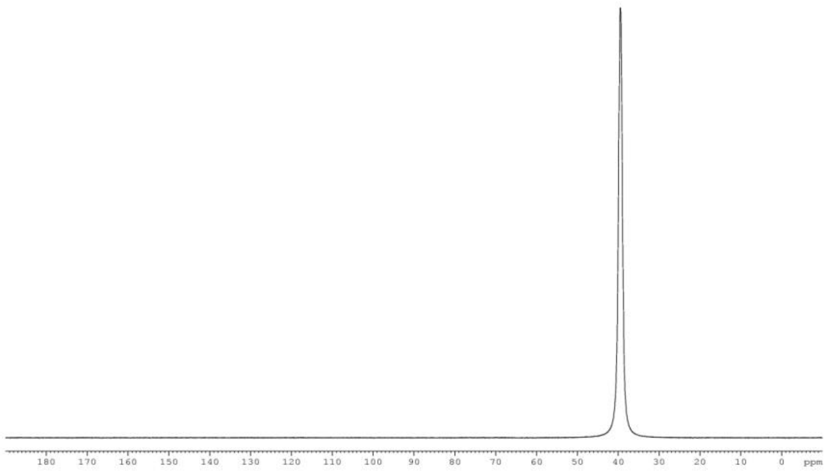

Figure S3. ^{13}C-NMR spectra of Mn(II)-Rut.

Table S1. Selected bond lengths (Å) and angles (°) between bonds in rutin and rutin–manganese complex molecules.

Atoms/rings	Rutin	Mn(II)/Ru
	**Bond lengths (Å)**	
**B**		
C1'-C2'	1.414	1.413
C2'-C3'	1.396	1.397
C3'-C4'	1.416	1.414
C4'-C5'	1.411	1.410
C5'-C6'	1.402	1.403
C6'-C1'	1.416	1.416
C3'-O3'	1.389	1.393
O3'-H3'	0.985	0.983
C4'-O4'	1.388	1.393
O4'-H4'	0.980	0.980
C1'-C2	1.468	1.468
C2'-H2'	1.080	1.079
C5'-H5'	1.089	1.089
C6'-H6'	1.083	1.083
**C**		
O1-C2	1.396	1.399
C2-C3	1.389	1.383
C3-C4	1.458	1.457
C4-C10	1.462	1.452
C10-C9	1.411	1.428

Commented [M1]: We have revised this Table number. Please confirm.

Commented [MK2R1]: I confirm

C9-O1	1.394	1.402
C4-O4	1.284	1.310
**A**		
C10-C5	1.426	1.446
C5-C6	1.396	1.417
C6-C7	1.414	1.398
C7-C8	1.406	1.412
C8-C9	1.395	1.388
C5-O5	1.385	1.360
O5-H5/Mn	1.023	1.881
C7-O7	1.389	1.397
O7-H7	0.979	0.979
C6-H6	1.086	1.087
C8-H8	1.083	1.083
O4-Mn	-	1.859
O5-Mn	-	1.881
	Angles (°)	
**B**		
C1'-C2'-C3'	121.562	121.627
C2'-C3'-C4'	119.692	119.834
C3'-C4'-C5'	118.971	118.839
C4'-C5'-C6'	121.193	121.234
C5'-C6'-C1'	119.939	120.009
C6'-C1'-C2	120.694	120.602
C2'-C1'-C2	120.786	121.042
C1'-C2'-H2'	120.194	119.981
H2'-C2'-C3'	118.238	118.387
C2'-C3'-O3'	120.792	120.655
C3'-O3'-H3'	108.950	108.527
O3'-C3'-C4'	119.516	119.511
C3'-C4'-O4'	117.694	117.881
C4'-O4'-H4'	112.119	111.642
O4'-C4'-C5'	123.333	123.279
C4'-C5'-H5'	119.223	119.208
H5'-C5'-C6'	119.574	119.553
C5'-C6'-H6'	120.076	120.214
H6'-C6'-C1'	119.984	119.777
**C**		
C1'-C2-C3	128.450	129.693
C1'-C2-O1	111.640	111.425
C2-C3-C4	121.646	121.840
C2-C3-O3	120.561	120.187
O3-C3-C4	117.688	117.801
C3-C4-O4	123.970	119.123
C3-C4-C10	115.596	117.966
O4-C4-C10	120.431	122.911

C4-C10-C9	121.035	118.432
C10-C9-O1	119.513	120.287
O1-C2-C3	119.900	118.877
C9-O1-C2	121.814	122.363
**A**		
C4-C10-C5	121.217	124.916
C10-C5-C6	120.960	119.591
C5-C6-C7	118.843	120.548
C6-C7-C8	122.032	121.502
C7-C8-C9	117.518	117.654
C8-C9-O1	117.587	115.693
C10-C5-O5	119.198	122.586
C5-O5-H5/Mn	107.690	123.942
O5-C5-C6	119.816	117.795
C5-C6-H6	119.334	117.601
H6-C6-C7	121.823	121.839
C6-C7-O7	121.880	122.551
C7-O7-H7	112.910	111.978
O7-C7-C8	116.087	115.946
C7-C8-H8	120.743	120.964
H8-C8-C9	121.738	121.379

[1]1Å = 10^{-10} m; [2]atom numbers according to Fig,

Table S2. NBO atomic charges[1] on atoms in molecules of rutin and its manganese complex

Atoms/rings	Rutin	Mn(II)/Ru
**B**		
[2]C1'	-0.121	-0.112
C2'	-0.246	-0.25
C3'	0.311	0.308
C4'	0.327	0.314
C5'	-0.28	-0.281
C6'	-0.182	-0.191
O3'	-0.721	-0.725
O4'	-0.702	-0.711
H2'	0.253	0.245
H3'	0.509	0.503
H4'	0.494	0.49
H5'	0.221	0.217
H6'	0.239	0.239
**C**		
C2	0.375	0.345
C3	0.185	0.195
C4	0.47	0.387
C10	-0.245	-0.232
C9	0.381	0.374
O1	-0.52	-0.537

Commented [M3]: We have revised this Table number. Please confirm.

Commented [MK4R3]: I confirm

O3	-0.589	-0.593
O4	-0.644	-0.635
**A**		
C5	0.398	0.395
C6	-0.341	-0.354
C7	0.39	0.374
C8	-0.309	-0.325
O5	-0.76	-0.772
O7	-0.714	-0.725
H5/Mn	0.528	0.642
H6	0.24	0.228
H7	0.501	0.493
H8	0.255	0.248

[1] 1e=$1.6021892 \cdot 10^{-19}$ C; [2] atom numbers according to Fig.
